# School readiness to adopt a school-based adolescent nutrition intervention in urban Indonesia

**DOI:** 10.1017/S1368980020001299

**Published:** 2021-06

**Authors:** Yessi Octaria, Apriningsih Apriningsih, Cesilia M Dwiriani, Judhiastuty Februhartanty

**Affiliations:** 1 Centre for Public Health Innovation, Udayana University, Denpasar, Bali 80232, Indonesia; 2 Southeast Asian Ministers of Education Organization Regional Centre for Food and Nutrition (SEAMEO RECFON)/Pusat Kajian Gizi Regional, Universitas Indonesia, Jakarta, Jakarta 104303, Indonesia; 3 Universitas Pembangunan Nasional “Veteran” Jakarta, Depok, West Java 16415, Indonesia; 4 Department of Community Nutrition, IPB University, Bogor, West Java 16680, Indonesia

**Keywords:** Schools readiness, Adolescent, Nutritional status, Diet

## Abstract

**Objective:**

To identify school community readiness to adopt a school-based adolescent nutrition intervention.

**Design:**

Cross-sectional study: mixed-methods design. The community readiness model was used to guide instrument development and qualitative analysis. Quantitative data are presented using descriptive statistics. Each statement was rated on a seven-point Likert scale, thereby producing scores between 1 (strongly disagree) and 7 (strongly agree).

**Setting:**

Ten of the twenty current public secondary schools in Bogor, Indonesia.

**Participants:**

Ninety teachers and ten school principals.

**Results:**

Eating behaviour problem awareness was present among all participants; awareness of efforts to improve eating habits was also present, but these efforts were perceived as having low efficacy; support from the City Education Authority and Health Authority was present, but the support type did not match the perceived needs; nutrition education had not been implemented across the entire school community due to competing priorities; existing nutrition policies did not provide concrete scenarios and clear guidelines for nutrition-friendly schools; the availability and accessibility of healthy foods at schools were considered to be key factors in improved adolescent nutrition; positive attitudes existed among respondents towards the implementation of various nutrition programmes, and the median and mode were seven in all types of school-based intervention.

**Conclusions:**

The school community readiness level regarding school-based adolescent nutrition interventions is currently in the action phase, implying that community leaders have begun organising efforts to address issues in adolescent nutrition and are aware of their consequences. Future support should be directed towards improving existing efforts and offering concrete ideas and clear policy guidelines for implementation.

As a developing country, Indonesia is experiencing a double burden of malnutrition, consisting of both under- and over-nutrition, both of which have long-lasting negative consequences for the quality of its public health, its resources and productivity^(1)^. Adolescent health and nutrition may represent new frontiers for endeavours to improve the quality of life of present and future generations and are emerging priorities in the national development agenda in this country^(2)^. The WHO defines adolescence as spanning the age range of 10–19 years^(3)^. According to this definition, approximately one-fifth of Indonesians are adolescents, 70 % of which currently attend school^(4)^. Thus, the school could be an effective setting to deliver nutrition interventions^(5)^.

Nutrition interventions in adolescents are believed to be essential for two main reasons. First, they may bolster the quality of life and educational achievements of adolescents by providing adequate nutrition during the periods of development and growth spurts. Second, it represents an investment for the future generation and may impart healthier habits onto this cohort as it moves into adulthood^(6,7)^. However, as with many other adolescent health interventions, implementing nutrition programmes can be expected to be more complex than similar interventions for younger children^(8)^. In 2013, the Indonesian Basic Health Research showed that 35·1 % of all adolescents aged 13–15 years old were stunted, while 11·1 % were underweight and 10·8 % were either overweight or obese. Furthermore, in regard to eating habits, the same report stated that 93·6 % of individuals aged 10 or over consumed an inadequate amount of fruits and vegetables, while more than half often consumed foods rich in sugar, fat and salt^(9)^. In addition, data from the Indonesian Global School Health Survey in 2015 showed that only one-third of students always had breakfast, only 3·81 % always brought their own food to school and more than half of teenagers consumed fast food at least once per week^(10)^. Therefore, to improve health promotion and prevention, the Indonesian government launched a national campaign to promote healthy lifestyles ‘Germas’ (Gerakan Masyarakat Hidup Sehat) in 2017^(11)^. However, widespread adoption of such a set of messages and its subsequent implementation as ‘a change’ in school settings requires readiness from the school community^(12,13)^.

Numerous school-based nutrition interventions have been conducted in Indonesia previously. The focus of attention was often directed at the type of media used for intervention, for example, the use of card games or puzzles aimed at improving knowledge in nutrition^(14–17)^. Meanwhile, studies to explore the potential of school communities as contexts and actors of change for the implementation of nutrition programmes have been rare. School communities are, in themselves, agents of change, with pivotal roles in sustaining a school environment that supports nutrition, health and learning. Hence, the supports from schools often determine whether an intervention may work in certain schools^(18)^. Given the current paucity of research on this topic, the current study aimed to identify the readiness of urban, public, secondary schools to adopt school-based adolescent nutrition intervention, explore the perceptions of teachers and principals regarding their school community readiness and identify opportunities for future improvements. These information might be used to facilitate the impact of school-based adolescent nutrition intervention.

## Methods

### Study design

**Fig. 1:**
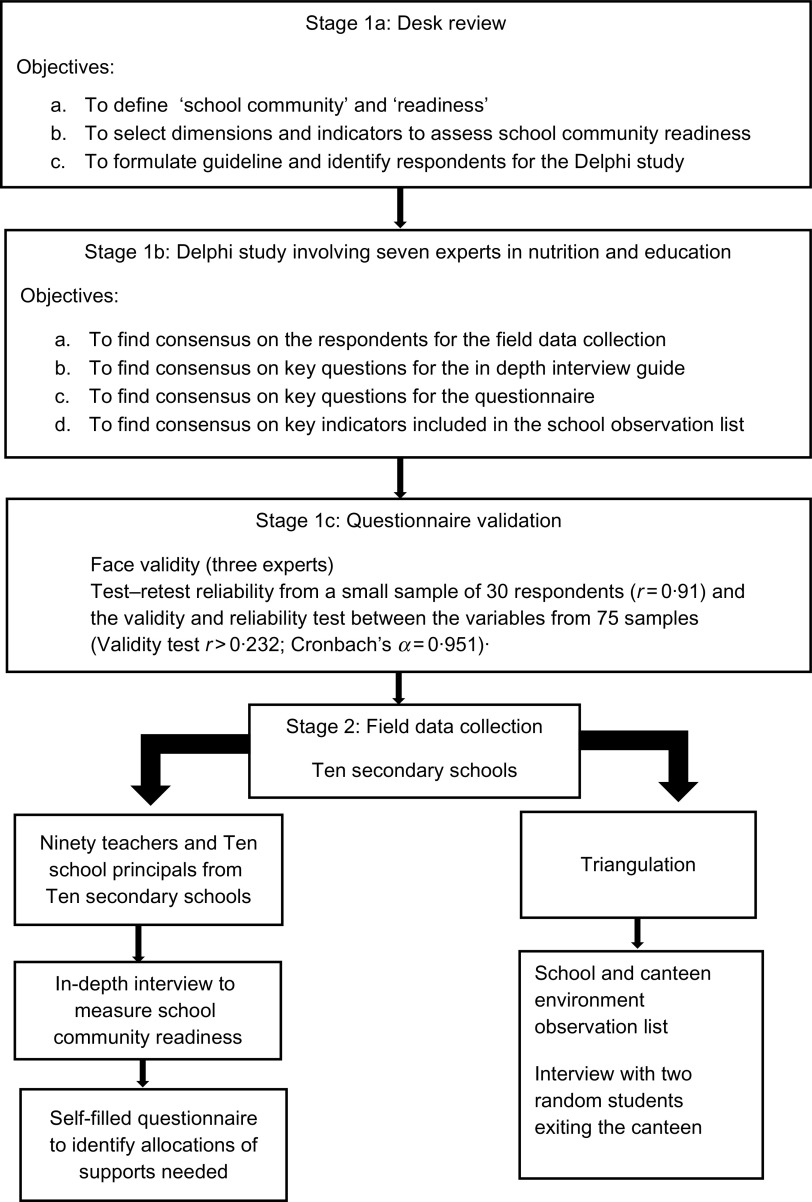
Research stages

A cross-sectional study with a mixed-methods design was conducted, involving two stages. The first stage consisted of instrument development, using two methods, that is, a desk review followed by a Delphi procedure involving seven experts in nutrition and education. The desk review was conducted to precisely define the concepts of ‘school community’ and ‘readiness’, thereby enabling the construction of dimensions and other indicators to assess readiness of school communities, and to determine respondents for the Delphi study. The desk review results informed the Delphi procedure through formulation of questions to enable expert consensus. The final outputs of this stage were instruments that could be used for field data collection, in particular the structured interview guide, self-administered questionnaires and an observation list as well as list of respondents. The second stage of the current study consisted of field data collection, which started with a short pre-structured interview by trained interviewers, followed by administration of self-questionnaires completed on the same day in the respondents’ workspace. The questionnaires passed face validity (three experts), test–retest reliability from a small sample of thirty respondents (*r* = 0·91) and validity and reliability test between variables from seventy-five samples (validity test *r* > 0·232, where 0·232 is the *r* table for seventy samples; reliability test with the Cronbach’s *α* = 0·951). The school environment was checked for availability of running water and soap for hand washing, the presence of trash cans in the classroom and school canteen and toilets, designated space to display adolescent health posters or other health-related information, as well as the availability of free drinking water. We also observed whether the school canteen sold healthy and nutritious foods such as fruit slices, vegetable dishes and bottled water as well as unhealthy snacks, for example, high-energy, low-protein snacks, deep-fried snacks and sweetened beverages. This school environment observation was done simultaneously with the interviews by trained enumerators (Fig. 1).

### Respondents and settings

The respondents for the current study included ninety teachers and ten school principals from ten secondary schools in the urban city of Bogor, West Java, Indonesia. The initial design was to involve both public and private schools; however, many of the private schools refuse to participate in the study. All public schools in Indonesia are mixed schools, with no separation for boys and girls. Experts in the Delphi study suggested a potentially effective mix of teachers who should be made responsible for conducting the nutrition education programme. This included the school principal, four classroom teachers, two science teachers, one physical education teacher, one counselling teacher and one art-and-culture teacher. Thus, from each school, one school principal and nine teachers were purposively designated as key informants by the research team. The ten schools were selected randomly from a total of twenty public secondary schools in Bogor using the Microsoft Excel 2013 random number generator to represent the readiness of the Bogor city school community.

### Data collection and data analysis

The in-depth interview guide was constructed based on the community readiness tool (CRT) developed by the Tri-Ethnic Centre for Prevention Research (Colorado State University)^(19,20)^. The CRT was designed for the planning and evaluation of complex, community-based health interventions^(21)^ but has also been used and validated in various community-based nutritional intervention studies^(22–24)^. The school CRT enables the assessment of six dimensions of readiness, providing a qualitative score based on nine stages of readiness^(25,26)^. The study was designed to help the Southeast Asian Ministers of Education Regional Centre for Food and Nutrition, in mapping the school communities readiness to adopt and build collaborative efforts for the school-based adolescent nutrition programme. Therefore, the research team made some adjustment to the CRT tool to serve the programme needs. The CRT adaptation, performed by the research team, was further validated by the Delphi procedure. The resulting, adapted tool is presented in Table 1.

**Table 1:** Semi-structured interview items

Questions
Which nutritional problems do you consider to be important to tackle in this school? Why do you think these problem(s) is (are) important? Are any efforts being made by the school to tackle the problem that you have identified? For how long have these efforts been made? Who is responsible for these nutrition-related efforts? Have the school principals been involved in these efforts? How are the school principals involved in these efforts (fund raising, management, assigning task force, etc.)? Are there special resources allocated for nutrition efforts in this school? Can you describe them? Does your school receive sufficient support (training, funds, monitoring and evaluation) from the city health authority? Was the support appropriate for your need? What do you need from them? Does your school receive sufficient support (training, funds, monitoring and evaluation) from the city education authority? Was the support appropriate for your need? What do you need from them? What are the barriers you experience in the implementation of the current nutrition-related efforts? What are your suggestions to improve nutrition programmes and activities for students in your school? What are your needs and concerns in this respect?

The initial CRT dimensions are community efforts, community awareness of the efforts, leadership, community knowledge about the issue, resources allocated for the issue and the community climate while the current study only assesses five dimensions, leaving out the community climate dimensions. A simplified, three-stage model of readiness was used, based on previous study by Martinez *et al.* ^(27)^ on community-based collaborative efforts. The adapted, three-stages scoring format is shown in Table 2. To ensure agreement in scoring, three independent raters read the qualitative result in order to create a consensus on the school community readiness level. The inter-rater agreement of scores in the five dimensions was calculated in IBM SPSS v24 (two-way mixed, average measures and absolute agreement), and the result was moderate with an interclass correlation average measure of 0·75 (*P* < 0·05).

**Table 2:** Readiness phase and criteria

Readiness phase	Suggested collaborative intervention
Phase 1: Pre-action	
Communities have little to no awareness of adolescent nutrition problem and eating behaviour problem. No efforts have been conducted to address the problem, and the community is not aware of ongoing efforts done, if any. No resources are allocated for the issue	Supply evidence based on the importance of nutrition in adolescence, advocate the issue of adolescent nutrition and eating habit, support efforts to identify and address issues in nutrition and eating behaviour at school and identify internal and external resources
Phase 2: Action	
The community and leaders are beginning to organise and conduct efforts related to adolescent nutrition and problematic eating behaviours. They have a sense of how nutrition problem in adolescent affects the school community. Some resources have been allocated for efforts of addressing the issue and building collaboration although adequacy and sustainability are still a problem. There is interest in partnership development of partnerships and mobilisation of the school community to address the issues of adolescent nutrition	Focus on supporting and/or facilitating ongoing efforts and to improve the quality of implementation. Initiate mobilisation and/or coalition development to increase support from identified internal and external stakeholders
Phase 3: Maintenance	
The community leaders have been engaged in continuous efforts to address issues regarding adolescent nutrition and eating habit. Community-wide initiatives involving all of the school elements may be underway. The school community has built partnerships and may have built name recognition for their adolescent nutrition programme and secured a funding stream	Focus on sustaining the quality of programme implementations. Consider rallying support from diversified coalitions and from new sectors. Support the dissemination of best practices and lessons learned from the school community to new communities for further scale-up

The structured in-depth interview guide was aimed to explore five of the six dimensions of community readiness, namely community efforts, community knowledge of the efforts, leadership, community knowledge about the issue of adolescent nutrition and resources allocated related to the issue, as well as the suggestions from respondents on how to improve nutrition interventions at their school. Each interview with teachers and the school principals was conducted by trained enumerators and lasted about 30–50 min. Semi-verbatim transcription notes from the interviews were grouped according to predetermined themes based on the community readiness dimensions and rated by an open code for identification of readiness level using a Microsoft Excel 2013 worksheet. All texts were written and processed in Bahasa Indonesia, and selected quotes were then translated into English for further use.

Following the in-depth interviews, respondents were given time to complete questionnaires on (i) the attitude regarding the importance of various nutrition-related activities to be conducted in their school, (ii) self-perceived capacity on knowledge, skills and dissemination of nutrition-related activities and (iii) the perceived leadership support. Teachers and school principals were asked to rate their level of agreement with statements related to nutrition interventions at their school, using a seven-point Likert scale ranging from 1 (strongly disagree) to 7 (strongly agree) for each statement.

The goal of the specially designed Likert scale questionnaires was to evaluate quantitatively the attitudes, self-perceived capacities and perceived leadership support of teachers and school principals towards nutrition-related efforts by the schools. These quantitative data were used to inform the design of collaborative intervention in more detail and to clarify where support should be allocated. Teachers and school principals were asked to rate their agreement to given statements on a graded seven-point Likert scale: 1 (strongly disagree), 2 (disagree), 3 (somewhat disagree), 4 (neither agree nor disagree), 5 (somewhat agree), 6 (agree) and 7 (strongly agree). Before the respondents completed the questionnaire, they were given a brief explanation and opportunity to skim through the questions and ask for clarification, if needed. The respondents then filled in the questionnaire individually at their own desk in the teachers’ room. Results were analysed using IBM SPSS v24 and are presented in descriptive statistics.

Data from each school were triangulated using the in-depth interviews of various teachers and school principals, questionnaire results and recorded observations from each school as well as from two randomly selected students exiting the school canteen during the observation. Thus, a total of twenty students consisting of four boys and sixteen girls were interviewed in all ten schools. Data were collected between March 2018 and August 2018.

## Results

### Respondents characteristics

The majority of teachers and school principals participating in the study were females (86·7 and 80 %, respectively). The age range of teachers was 22–67 years (mean 49 years), with 85 % of all teachers being aged >40 years. For school principals, the age range was 49–58 years, with an average age of 52·3 years (Table 3).

**Table 3:** Characteristics of the key informants

Teachers	*n*		%
Age			
Mean		49	
Min–max		22–67	
Female	78		86·7
Male	12		13·3
Total	90		100
School principals			
Age			
Mean		52·3	
Min–max		49–58	
Female	8		80
Male	2		20
Total	10		100

### Qualitative results

#### Community knowledge about the problem of adolescent nutrition

The in-depth interviews with teachers and school principals revealed that the majority of respondents were unaware of problems in nutrition status such as thinness (i.e. having a body weight less than their peers of the same age or height), obesity (having a body weight more than their peers of the same age or height), stunting (being shorter than their peers of the same age) and anaemia (having low Hb or showing any sign of fatigue, weakness or paleness) as demonstrated by the quote below:

I don’t think those are problems for us. Students from the first year are usually shorter and smaller because they just graduated from elementary school…I think body shape is genetic and we need to be very careful talking about it. (Female Science Teacher 1, School 7)

However, they are well aware of unhealthy eating habits among adolescents as well as their consequences.

They often skip breakfast, and they don’t eat enough fruits and vegetables. They snack on a lot of unhealthy food and don’t drink enough water, more often drink cold sweet drinks, especially sweet iced tea (Female Classroom Teacher 3, School 6)

Breakfast, that’s an important problem. The school starts early at 7 am, so many of the students do not have time for breakfast. Mind you, the school now, due to the new curriculum, ends at 2.30 pm so that they have to eat two meals during school hours. I think what they eat at school is important. This will affect their school achievement and health. (Female School Principal, School 2)

#### Community efforts and community knowledge of the efforts

Teachers, school principals and students were asked about what kind of nutrition-related activities have been conducted how often and for how long in their school. All schools cited they had a breakfast programme, which involved students bringing their packed breakfast from home and eating it together at school. This was part of the city government directive to run a breakfast programme in all schools. However, the exact implementation varied between schools, with some schools conducting the breakfast programme regularly and some only running them on certain important dates, that is, two or three times per semester. The quality of the implementation also differed widely, with some schools reporting that classroom teachers or school principals usually inspect the food and give simple explanations with regard to eating a varied diet and the importance of eating vegetables and fruits, while other schools were just eating together without much elaboration, as evident from the following quotes:

We have a breakfast programme every Friday, well, not every Friday but at least twice a month either in the school yard or in the classrooms. We encourage them to bring healthy food, with vegetables, fruits and a protein source. Sometimes, our school principal would check the food, sometimes the classroom teacher does it, and we encourage students to share their food with their friends. (Female Classroom Teacher 3, School 6)

We have a breakfast programme, but we rarely do it. It was just eating together. Many of the students bring fried food like fried sausages or nuggets, no vegetables, but what can we do about it? At least it is good that they have something for breakfast. Some forget to even bring food, so they buy it from the canteen. What is the point then? (Female Science Teacher 2, School 7)

Aside from the breakfast programme, other programmes mentioned by teachers and school principals were focused on healthy school canteen. However, interviews with teachers and school principals as well as other data indicated that measures to improve the health standards in school canteens were limited to rules for food safety and physical facilities, such as availability of trash cans, hand-washing spots and cleanliness. Current official regulations for healthy school canteens, such as the Healthy School Canteen Guideline (issued by the Ministry Education in 2014) and the National Guideline for Child-Friendly Schools (issued by the Ministry of Women Empowerment and Child Protection in 2015) do not include regulations regarding sales of nutritious foods from varied sources. This was confirmed by our observation that only two of the ten examined schools sold sliced fruits, and none sold dishes with sufficient amounts of vegetables. In contrast, all schools sold sweetened beverages and high-energy fried snacks.

School teachers and principals were asked, ‘Have the school principals actively looked for funding and involved in the implementation of school-based nutrition programmes?’, ‘Is there any task force or have specific individuals been appointed to deal with these matters?’ and ‘How are the school-based nutrition programmes of this school being managed?’. All interviewed teachers declared that their school principals had been very supportive of any programme instructed by the government, including those related to breakfast and school canteens, which were considered to be nutrition related by the respondents. All schools were said to have their own appointed persons who were responsible for the breakfast programme. In some schools, this responsibility was assigned to the classroom teachers, while in other schools, the school principal managed the programme. Regarding the allocation of funding, the respondents acknowledged that the breakfast programmes essentially did not require any specifically allocated funding.

Regarding the school canteens, in each school the canteen space is rented to private tenants. However, all schools also had a committee that manages the canteen. Two schools had stricter rules and regulations for the canteen, including regulations for the kinds of food and snacks allowed, food handling and cleanliness of the canteen. Other schools had a more relaxed approach to canteen regulations. With regard to the budget sources for canteen maintenance, both teachers and school principals stated that their canteen required continuous financial support, and that funding nowadays derives mostly from the rent paid for the canteen space and government funding, as strict regulations preclude gathering donations from parents. Thus, selling some items that are profitable has become the main concern for tenants while nutritional value of foods receives less focus.

#### Barriers for implementation

Teachers and school principals stated that an important barrier to effectively implementing nutrition interventions was their lack of time to attend trainings outside the school; consequently, only one specific teacher was commonly sent to attend many training sessions related to health and nutrition. These were often the individuals with the fewest teaching hours, such as the counselling teacher. However, because of their limited number of teaching hours they typically have the least contact with students. The respondents stated that involving more teachers would be an important step to scale up nutrition interventions and to improve the sense of ownership of the programme at school:

It is better to involve all teachers for the technical guidance on how to implement nutrition programme at school, so that all teachers can participate in the program (Female English Teacher/Classroom Teacher, School 4)

Respondents suggested that teacher training within the schools could be good approaches to improve the number of teachers receiving nutrition education, so that more teachers can contribute to nutrition programmes. Another barrier that became evident was the lack of simple and clear guidance from the local health authority on how to conduct nutrition programmes and to create school environments that promote good nutrition. A mismatch was noticed between the information given during training and the actual needs of schools. For example, the training provided teachers with knowledge regarding balanced nutrition while what the teachers actually needed were ways to translate that knowledge into menus or foods to be sold by the canteen. In addition, the lack of means for effective communication with canteen tenants was also mentioned as a barrier in several schools.

#### The way forward

Many suggestions for future improvement in school-based adolescent nutrition programmes gathered from the interviews highlighted practical need for concrete examples of healthy and nutritious foods.

…just do the concrete stuff….the example of what we should eat, like the menu and the amount of food we need to eat, if the students want to bring a packed lunch, what should it look like? (Male Science Teacher 2, School 9)

….the breakfast programme, it is good… but all we know is if it is not healthy or not varied enough…but without concrete examples of what is considered nutritious, how will they improve? (Female Classroom Teacher 3, School 1)

The school principals weighed in on the importance of healthy school canteens and how to increase the availability of healthy and nutritious food, while maintaining the affordability of the food on offer, without hindering affordability of foods and revenue of the canteens.

I have restricted them (canteen tenants) from selling this and that, such as food with artificial colouring, taste enhancers, artificial sweeteners, and preventing them from using plastic or Styrofoam cups or bowls, and they (canteen tenants) have followed the rules. I also raised the topic of selling nutritious food during some meetings with them, but they asked me some suggestions on what to sell. I didn’t know the answer, so what I need is answers to these questions. What kind of food should be sold in the canteen that is nutritious, tasty, and not to expensive such that the student can afford it and still profitable…the university should help me with this. (Female School Principal, School 6)

The school principals also mentioned a need for clear guidelines for healthy nutrition at schools and a checklist or tools that can be used for self-evaluation on their progress.

### Quantitative results

Teachers and school principals both indicated positive attitudes towards and mostly strong agreement with the importance of various school-based nutrition interventions in their school to stimulate fruit and vegetable consumption, water intake, reduction of sugar, salt and fat intake, improving breakfast habits and increasing physical activity among students. This was reflected by very high score (median and modes of 7) in the Likert scales of both principals and teachers for each of the relevant statements (Table 4). However, the strongest agreement was found for the programme to improve the consumption of fruits and vegetables. Ninety percent of the school principals and 70 % of teachers were in favour of this type of intervention, followed by intervention to improve water intake and breakfast habits which were supported by 70 % of both the school principals and teachers. However, teachers reported less agreement than school principals with statements expressing readiness to be involved in training, readiness to implement the skills acquired through training and ability to ask peers and parents to actively help solve nutrition problems. This was reflected by lower median scores on these statements among the teachers than among the school principals (Table 4). In addition, school principals reported higher self-perceived readiness (median score 6·5) to lead the activities to solve nutrition problem in their school, than did teachers (median score 5). Fifty percent of the school principals opted for ‘strongly agree’ to the statement compared with only 22·2 % of the teachers.

**Table 4:** Attitudes towards the importance of nutrition intervention, self-perceived capacity, and perceived leadership support

Score	School principals (*n* 10)			Teachers (*n* 90)		
	Median	Mode	Range	Median	Mode	Range
Statement on attitude towards nutrition-related intervention						
Activities to increase students’ fruit and vegetable intake are needed in this school	7	7	6–7	7	7	5–7
Activities to increase students’ protein intake are needed in this school	7	7	5–7	7	7	5–7
Activities to increase students’ water intake are needed in this school	7	7	5–7	7	7	4–7
Activities to reduce students’ salt, sugar and fat intake are needed in this school	7	7	5–7	7	7	4–7
Activities to improve students’ breakfast habit are needed in this school	7	7	5–7	7	7	5–7
Activities to increase students’ physical activity are needed in this school	7	7	5–7	7	7	5–7
I am ready to participate in nutrition training to improve my knowledge and skills to solve nutrition problems in this school	6·5	7	5–7	6	7	3–7
I am ready to implement the acquired knowledge and skills I received by training to solve nutrition problems in this school	7	7	5–7	6	7	3–7
I am ready to ask my peers and parents to implement activities to solve nutrition problems in this school	7	7	5–7	6	7	3–7
I am ready to lead the activities to solve nutrition problems in this school	6·5	7	4–7	5	5	3–7
Statement on self-capacity						
I have adequate knowledge about balanced diets	5	5	3–7	5	5	3–7
I have adequate knowledge about healthy and hygienic lifestyles	5·5	5	3–7	5	5	3–7
I have adequate knowledge about high-salt, high-sugar and high-fat diets and their consequences	5	5	3–7	5	5	3–7
I have adequate skills to promote balanced diets	5·5	5	4–7	5	5	1–7
I have adequate skills to promote healthy and hygienic life styles	5	5	5–7	5	5	1–7
I have adequate skills to promote low-salt, low-sugar and low-fat diets	5	5	5–7	5	5	1–7
I have adequate skills to advocate stakeholders to support implementation of nutrition interventions in this school	5·5	5*	4–7	5	5	1–7
The school has adequate human resources to implement school-based nutrition interventions	5·5	5*	4–7	6	6	3–7
The school has adequate resources to implement school-based nutrition interventions	6	6	3–7	6	6	3–7
Statement on leadership support						
The school principal has given the implementation of nutrition intervention in this school sufficient support	6	6	5–7	7	7	4–7
My fellow teachers have given the implementation of nutrition intervention in this school sufficient support	6·5	7	4–7	6	7	4–7
The local education authority has given the implementation of nutrition intervention in this school sufficient support	5·5	5*	4–7	6	6	4–7
The local health authority and the community health centre have given the implementation of nutrition intervention in this school sufficient support	5·5	4*	4–7	6	6	4–7
The support given by the local education authority has been appropriate for the school’s needs	5	7	4–7	6	6	3–7
The supports given by the local health authority and the community health centre have been appropriate for the school’s needs	5	4*	4–7	5	5	3–7

* Where there were two modes, the lower mode was reported.

The majority of teachers and school principals answered ‘somewhat agree’ (score 5) to self-statements about having adequate knowledge about balanced diet, healthy lifestyle and excess consumption of sugar, salt and fat. Similar scores were obtained for statements regarding the possession of skills to promote balance diet, healthy and hygienic lifestyles and to reduce sugar, salt and fat consumption among students. Majorities of both teachers and the school principals answered ‘somewhat agree’, as reflected by the scores for these statements which mode was 5 (Table 4). Around 40–50 % of teachers answered ‘somewhat agree’ to these statements, while for the school principals, the proportion of answering somewhat agree was 30–50 %.

Both teachers and school principals responded more affirmatively to the statement that their school had sufficient non-human resources to implement nutrition interventions, as shown by median scores of 6 (agree; Table 4). Forty percent of the school principals answered ‘fairly agree’, while 50 % of teachers answered the same. In contrast, teachers had higher scores than school principals when responding to the statement that their school had adequate human resources to implement nutrition programmes. The median and mode scores were 6 (agree) for the teachers and 5·5 and 5 for the school principals, respectively (Table 4).

For leadership support, teachers strongly agreed with the statement that the school principal had provided sufficient support for the implementation of healthy lifestyle at their school. However, the school principals voiced a slightly different opinion and most often rated ‘fairly agree’ to this statement. Regarding support from fellow teachers, the majority of both teachers and principals responded ‘strongly agree’, implying that they experienced sufficient support from their colleagues. For external support, teachers rated the support from the education authority and the health authority as ‘fair enough’ (rated 6), while the school principals rated the adequacy of support lower, with 30 % of the school principals answering ‘somewhat agree’ (rated 5) to the statement that the City Education Office had provided sufficient support, and 30 % answered ‘neither agree nor disagree’ (4) that the City Health Office has provided sufficient support. Regarding the appropriateness between support given by the Education and the Health Authority and the schools’ needs, support from the City Education Office was rated as more appropriate than the support from the City Health Office, with the school principals scoring lower than teachers on both statements.

## Discussion

The current study explored the readiness among the Bogor city school community to adopt a school-based adolescent nutrition intervention. The working definition of readiness in the current study, based on work by Weiner *et al.* ^(28)^, was the level of preparedness of the school community to adopt change, that is, to embrace and implement a school-based adolescent nutrition programme. The data from our study showed that many school communities were unaware of issues in nutrition status such as stunting, thinness or overweight and obesity. However, the community was well aware of issues regarding eating behaviour, including skipping breakfast, inadequate fruit and vegetable intake and unhealthy snacking. The community was also generally aware of the impact of eating issues on school achievements and health in adolescents.

Programmes related to nutrition were already ongoing at schools, such as the breakfast programme and striving to attain healthy school canteens. The school communities were well aware of these ongoing efforts. As community leaders, school principals were found to be supportive of these efforts and had allocated time and human resources for these efforts. Thus, qualitative assessment based on the community readiness model indicated that the Bogor city school community readiness was in phase 2, implying that the communities and leaders were aware of the issues and their impact, efforts had been made and registered by the community. In addition, commitment in terms of resources allocation was also present (Table 2). Given this state of affairs, collaborative efforts should be directed towards supporting ongoing efforts to address issues of adolescent nutrition and eating behaviours both from within the school community and from external relevant stakeholders by building effective coalitions. The next questions were how to best support the school community in their existing efforts, and how to build effective coalitions for the school community to improve their efforts.

The readiness factors are defined as any practice or characteristic that aids the organisational transformation by eliminating possible barriers for success or providing the knowledge and capabilities required to succeed in establishing change^(29)^. Our study showed that, despite the ongoing efforts and resource allocations for addressing adolescents’ nutrition issues by schools, many thought that the programmes lacked the efficacy to actually improve eating habits among students. The barriers for implementation identified in the current study included the absence of concrete policy guidelines to achieve nutrition-friendly schools, the lack of time of teachers to attend relevant training, lack of food-related knowledge and know-how and communication barriers with the canteen vendors.

The problem of lacking practical contextual guidelines and indicators has also been cited in other studies^(30,31)^. A study on school wellness policy in the USA showed that a higher quality of the written policy was associated with more effective implementation of health-promoting practices at the school level^(32)^. Moreover, a study in Mexico suggested that continuous monitoring of national policies is important to ensure quality of its implementation at the local level^(33)^. Hence, in order to improve school-based nutrition programmes in Bogor city, the school community would need a more comprehensive written policy with clear requirements and indicators. Systems to monitor and evaluate implementation of policies should be in place for quality insurance, and these systems should include self-assessment mechanisms to map progress. Such approach has been proposed in the WHO/UNICEF/FAO Nutrition-Friendly School Initiative^(34)^. The Nutrition-Friendly School Initiative was piloted in sixteen countries; it uses an accreditation scheme which assesses five core components, namely nutrition-friendly school policy, capacity building and awareness campaign, nutrition-related curriculum, as well as creating supportive school environment and school-based nutrition services^(35)^. The Nutrition-Friendly School Initiative pilot projects assessment in Burkina Faso and Benin showed improvement in the school and the community mobilisation towards improved nutrition due to the initiative^(36)^.

Our study also highlighted another important point, namely that environment change is pivotal when trying to improve the practice of healthy eating among adolescent students. The respondents suggested that improving availability and securing access to healthy and nutritious foods via the school canteen represent key elements for the school nutrition programme, and that communication with the canteen vendor is therefore essential. As previous studies have shown, increased availability of healthy foods improves healthy food choices and nutritional state among adolescents and young adults^(37–39)^. The communication problem with canteen vendors could partially be solved by addressing the food literacy issue. Our respondents reported a lack of concrete examples of healthy menu items for teachers, students and food vendors which could be used as reference, and that this lowers their self-efficacy when trying to deliver nutrition education and to provide more healthy food choices. These results are especially relevant because previous studies have shown that improvements in food literacy and planning do improve healthy food intake of fruits and vegetables among adolescents and young adults^(40,41)^. Therefore, we propose that the nutrition education modules for schools should include more practical skills such as food literacy and food preparation.

This touches on the problem of the shortage of time to attend training. As shown by quantitative scores from the self-descriptive Likert scale questionnaires from our study, our respondents considered themselves ‘neutral’ in terms of their knowledge level and skills to implement nutritional programmes, which was in stark contrast to the importance they attributed to the various nutrition interventions at their school. One US study found that lack of nutritional knowledge among teachers was not correlated to the actual implementation of nutrition programmes^(42)^. However, multiple studies have reported that lack of knowledge and skills for implementation of programmes by school staff could be regarded as a barrier for programme implementation in developing and developed countries alike^(43,44)^. Our respondents suggested that nutrition training for staff members be conducted at their schools, thus allowing more teachers to attend and be educated on adolescent nutrition to develop and improve their relevant knowledge and skills. Another possible solution might be to offer online training. Availability of flexible training times and having a source of trusted knowledge on nutrition continuously available may be less burdensome than physically attending the course. On-going support in the form of knowledge and skills resources as well as tailored feedback via text messaging to the school queries following training has been identified as an effective approach^(45)^. Since 2017, the Southeast Asian Ministers of Education Regional Centre for Food and Nutrition has developed an online training for teachers as part of its Nutrition Goes to School programme. The training uses a blended platform comprising a Massive Open Online Course approach and WhatsApp group that is moderated by trained nutritionists. The training was approved by the Ministry of Education, and teachers who completed this training are rewarded with professional credit units which they can use in their accreditation. We view approaches such as this as an opportunity to foster the effectiveness of nutritional interventions in the junior high school community in Bogor city.

The individual perspective of both teachers and school principals showed a positive attitude towards the implementation of various nutrition interventions in their school, indicating general support across the school community. This is especially encouraging because a major barrier for implementation of various school-based nutrition programmes was the alignment of optimal nutrition with the schools’ priorities^(30)^. In addition, the school principals themselves expressed great willingness to attend training and to use their gained nutritional knowledge and skills to realise their efforts. This is positive outcome, since the principals could be considered as the schools’ gatekeepers with the power to facilitate the adoption and implementation of school-based nutrition programmes. However, another outcome of our study was that support from the health and education authorities albeit adequate did not match the school needs. Notably, the type of trainings given did not effectively improve food literacy, the training did not stimulate the involvement of more teachers, there was a lack of clear policy guidelines to implement, for example, the breakfast and healthy school canteen programmes and finally an absence of continuous support and effective monitoring and evaluation tools for the programmes. Our respondents also pointed out the ineffective communication with canteen tenants to improve the quality of food sold as an important barrier for adolescents to change their eating behaviours. Developing a simple system to rank food from nutrition and health perspectives is generally accepted as an effective approach towards limiting sales of unhealthy food in various settings, in both developing and developed countries; a notable illustration of such an approach is the use of the traffic light system and its variants^(32,33,42)^. This has bearing on our findings that food literacy training and clear policy guidelines were absent in Indonesia. Therefore, endeavours to strengthen external leadership should include the practical development of nutritional literacy and the development of clearer policy guidelines and indicators. The collaboration between academia, and local education and health authorities is needed to overcome these hurdles by ensuring continuous technical support and coaching for the school to achieve more success for school-based nutrition programmes. However, one should also be aware that the pursued effects of interventions may be rather slow to develop as illustrated by a study from the USA. The study showed that whereas after 3·5 years the nutrition intervention had resulted in better policy implementation, significant changes in water intake and lower consumption of fruit juice but had not yet produced significant effects on students’ other eating habits or body weight^(46)^.

## Strengths and limitations

The current study is the first in Indonesia to map out the school community readiness for school-based nutrition intervention aimed at adolescents. Also, its combined qualitative and quantitative outcomes provide insights into the current readiness for nutritional programmes and the types of support needed by schools to make them successful. However, our study also has several limitations that warrant discussion. The variety of schools that participated is limited in the sense that they are all public schools that may show higher acceptance towards government-initiated programmes. Therefore, to gain a wider perspective, more school types, for example, private schools with children from various socio-economic backgrounds, and religion-based schools, including religion-based boarding schools should be involved in future studies. The decision to involve only teachers and school principals as respondents in our study was based on lengthy, internal deliberations. In the Indonesian context, the stakeholders for a nutrition programmes are often not clearly defined. For studies that have defined school principals or teachers as stakeholders, the selected range individuals were often restricted, only to science teachers, school health teachers or principals, rather than representing the whole school. Another limitation was that parental involvement in school programme decision making in Indonesian public school tends to be very limited. Therefore, if the intervention purports to strengthen the agency of schools to implement a school-based nutrition programme, the definition of school community should be clearly restricted to teachers and school principals, before addressing the role of the wider school community, including the students and their parents.

## Conclusions

The school community readiness level in Bogor, Indonesia was found to be in the action phase, implying that community leaders are aware of the impact of adolescent nutrition problems and have begun organising efforts to address these issues. In addition, community members are also well aware of these issues. Future support should be focused on improving the ongoing efforts as well as intensifying the development of coalitions. The barriers for implementation identified by the respondents included the lack of time to attend training, lack of clear and practical guidelines, mismatch between the offered support and the schools’ most important needs and ineffective communication with canteen tenants regarding improvements of the nutritional value of the offered food. The primary nutrition-related community interests are to increase consumption of vegetables and fruits, drinking more water and to improve breakfast habits. Modules should be prepared to educate teachers and canteen tenants. The content should improve food literacy by providing a sample menu (food, dishes and serving size) and nutritious food selection, as well as the directions to prepare these foods. Given the limited time available for training, mixed-media content delivery, for example, via online training and text-message group support would be most optimal. An additional need exists to develop clearer and simpler policy guidelines for the school community, including checklists for nutrition-friendly schools with guidelines to promote healthy nutrition in canteens and traffic lights system for offered food items based on nutritional content. Subsequent studies should involve a greater diversity of schools.
